# Surfing on Fitness Landscapes: A Boost on Optimization by Fourier Surrogate Modeling

**DOI:** 10.3390/e22030285

**Published:** 2020-02-29

**Authors:** Luca Manzoni, Daniele M. Papetti, Paolo Cazzaniga, Simone Spolaor, Giancarlo Mauri, Daniela Besozzi, Marco S. Nobile

**Affiliations:** 1 Department of Mathematics and Geosciences, University of Trieste, 34127 Trieste, Italy; 2 Department of Informatics, Systems and Communication, University of Milano-Bicocca, 20126 Milano, Italy; d.papetti1@campus.unimib.it (D.M.P.); simone.spolaor@disco.unimib.it (S.S.); giancarlo.mauri@unimib.it (G.M.); daniela.besozzi@unimib.it (D.B.); 3 Department of Human and Social Sciences, University of Bergamo, 24129 Bergamo, Italy; paolo.cazzaniga@unibg.it; 4 Department of Industrial Engineering & Innovation Sciences, Eindhoven University of Technology, 5612 AZ Eindhoven, The Netherlands

**Keywords:** global optimization, particle swarm optimization, fuzzy self-tuning PSO, Fourier transform, surrogate modeling

## Abstract

Surfing in rough waters is not always as fun as wave riding the “big one”. Similarly, in optimization problems, fitness landscapes with a huge number of local optima make the search for the global optimum a hard and generally annoying game. Computational Intelligence optimization metaheuristics use a set of individuals that “surf” across the fitness landscape, sharing and exploiting pieces of information about local fitness values in a joint effort to find out the global optimum. In this context, we designed surF, a novel surrogate modeling technique that leverages the discrete Fourier transform to generate a smoother, and possibly easier to explore, fitness landscape. The rationale behind this idea is that filtering out the high frequencies of the fitness function and keeping only its partial information (i.e., the low frequencies) can actually be beneficial in the optimization process. We prove our theory by combining surF with a settings free variant of Particle Swarm Optimization (PSO) based on Fuzzy Logic, called Fuzzy Self-Tuning PSO. Specifically, we introduce a new algorithm, named F3ST-PSO, which performs a preliminary exploration on the surrogate model followed by a second optimization using the actual fitness function. We show that F3ST-PSO can lead to improved performances, notably using the same budget of fitness evaluations.

## 1. Introduction

Most optimization problems, related to real-life applications, are characterized by the existence of a large number of local optima, which might induce a premature convergence of optimization methods and prevent the identification of the global optimum. If we consider this issue from the perspective of a search on the fitness landscape, we are forced to face the exploration of a rugged multidimensional surface, which makes the search for the global optimum pretty hard and time consuming. In order to facilitate optimization tasks, we metaphorically take inspiration from the fact that surfing in rough waters is arduous and annoying, while riding a big wave is much easier and more fun. Similarly, in this work we aim at simplifying the optimization task by smoothing the fitness landscape, i.e., getting rid of (a number of) local optima thanks to the generation of a surrogate fitness surface.

In the field of optimization, surrogate modeling consists in the definition of an approximated fitness function, whose evaluation is typically less expensive than its original counterpart [1]. Commonly employed techniques include polynomial regression [2], Kriging modeling [3], support vector regression [4], radial basis functions [5], artificial neural networks [6], genetic programming [7], or a combination of the above, employed to build local or global surrogate models [8,9,10,11].

The construction of surrogate models is exploited in several engineering and scientific fields [1,12,13,14,15], to lessen the computational burden of optimization tasks in which the evaluation of the fitness function is a major bottleneck. Indeed, many different optimization problems typically require the execution of time-consuming computer simulations for each evaluation of the fitness function, like, for instance, engineering design, drug design, and biochemical simulation. Leveraging surrogate models proves particularly useful when the optimization problems are tackled by means of evolutionary computation and swarm intelligence algorithms [16,17,18], which require a huge number of fitness evaluations to converge to optimal solutions. Besides the reduction of the computational cost, surrogate modeling has been used to address other issues [16], e.g., searching for optimal solutions that are insensitive to small perturbations [19]; learning changes in the fitness landscapes in dynamic optimization problems [20]; smoothing noisy or multimodal fitness functions [21,22].

Following this line of research, here we present a novel method, named surF, which leverages the direct Fourier transform to generate smoothed surrogate models of multimodal, rugged, and noisy fitness landscapes. In addition to the advantage of creating surrogate fitness landscapes that are computationally less expensive to evaluate, surF provides the user with the possibility of tuning the “level of smoothing” by means of a hyperparameter, which corresponds to the number of low frequency spectral coefficients considered for the inverse Fourier transform. To assess the usefulness of surF in facilitating the convergence to the global optimum, we test our approach on a set of benchmark functions characterized by ruggedness, noise, and multimodality.

**Figure 1:**
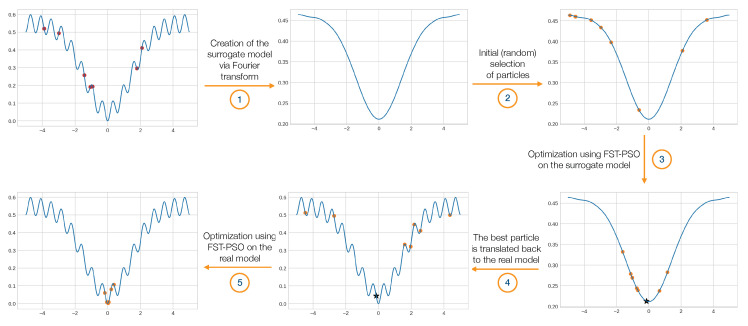
F3ST-PSO phases. *Step 1*: surF randomly samples the fitness landscape within a chosen search space (red dots) and uses that information to create a surrogate and smoother model. *Step 2*: a population of random candidate solutions is generated and placed on the surrogate model of the fitness landscape (orange dots). *Step 3*: FST-PSO is exploited to perform an optimization on the surrogate model. *Step 4*: the best individual (black star) found by FST-PSO is placed on the original fitness landscape, together with a new population of random candidate solutions (orange dots). *Step 5*: a final optimization with FST-PSO is performed on the original fitness landscape.

In this work, we thoroughly investigate the efficacy and effectiveness of surF, by coupling it to FST-PSO [23], a settings-free version of Particle Swarm Optimization (PSO) [24]. FST-PSO was specifically chosen among the swarm intelligence techniques since it is a self-tuning algorithm: as a matter of fact, the lack of any hyperparameters simplifies the analysis of surF’s performance, which could be otherwise affected or even covered up by the selection of the optimizer’s functioning settings. The surrogate model created by surF is directly exploited by FST-PSO to perform a (computationally less expensive) optimization on a smoother fitness landscape. This idea represents the foundation for F3ST-PSO, a novel dual-phase variant of FST-PSO that begins the optimization on the surrogate model and terminates on the real fitness landscape (Figure 1).

F3ST-PSO starts by randomly collecting samples of the fitness landscape in a given search space: this information allows surF to create a surrogate model using Fourier transforms and filtering (step 1). Then, a random population of candidate solutions is initialized on the surrogate model (step 2) in order to perform a FST-PSO optimization (step 3); the best particle found in the surrogate model is then identified and translated to the original fitness landscape (step 4), where it provides a hint for a new optimization with FST-PSO using the real model (step 5).

F3ST-PSO was applied for the optimization of a set of classic benchmark functions and the CEC 2005 test suite. We show that, in general, the application of F3ST-PSO allowed us to obtain better results than FST-PSO, thanks to the surrogate models created by surF, which effectively remove noise and local optima that might impair the convergence process.

A detailed description of the creation of the surrogate model by the surF algorithm, and its coupling with FST-PSO, is provided in Section 2, while in Section 3 we show that F3ST-PSO can outperform FST-PSO, especially in the case of very noisy fitness landscapes. Finally, in Section 4 we draw some conclusions and provide some insights on future developments of this line of research.

## 2. Materials and Methods

Given a *D*-dimensional search space $S\subseteq R^{D}$ and a fitness function $f:R^{D}\to R$, a minimization (maximization) problem consists in finding the optimal solution o∈S such that f(o)≤f(x) (f(o)≥f(x)), for all x∈S,x≠o. Since the fitness function associates each candidate solution in the search space to a real value, it induces a hypersurface representing the quality of solutions across their feasible space of existence. This surface traditionally takes the name of fitness landscape, and can be formally defined as a pair Λ=(S,f). It is often the case that these hypersurfaces are irregular, rugged, noisy, funnelled, and so forth. This circumstance prevents the adoption of simple approaches like hill climbing and gradient descent to determine the optimal solution, since these methods would easily get stuck in a local minimum. To bypass this issue, a plethora of metaheuristics (e.g., stochastic gradient descent, simulated annealing, and the methods belonging to evolutionary computation and swarm intelligence) have been proposed and proved to be effective on a large number of benchmark functions and real applications. In the next sections, we first recall one advanced metaheuristic for global optimization, we introduce a novel approach to smooth the fitness landscapes by means of Fourier transforms, and finally show how to couple these approaches to carry out efficient optimization on surrogate models of the fitness landscape.

### 2.1. Fuzzy Self-Tuning PSO (FST-PSO)

Particle Swarm Optimization (PSO) is a population-based metaheuristics for global optimization inspired by the collective movement of fish and birds [24]. In this algorithm, a set (swarm) of *P* candidate solutions (particles)—each one identified by a position vector $\vec{x}$—moves inside a bounded *D*-dimensional search space. Particles cooperate to identify and converge to the (global) best solution of the optimization problem. In the classic formulation of PSO, the balancing between the global exploration and the local exploitation of particles is determined by two specific settings: the cognitive attractor $C_{\mathrm{cog}}\in R^{+}$ and the social attractor $C_{\mathrm{soc}}\in R^{+}$, respectively. These two factors have a relevant impact on the performances of PSO, along with the inertia factor $w\in R^{+}$ that is used to prevent a chaotic movement of particles. On top of that, the values of the maximum and minimum velocity (${\vec{v}}_{\max},{\vec{v}}_{\min}\in R^{D}$) of particles along each dimension of the search space can also affect the quality of the optimal solutions found by PSO: the former prevents extreme movements inside the search space (possibly leading the particles outside the feasible region), while the latter prevents the stagnation of the swarm. Due to their importance, $C_{\mathrm{soc}},C_{\mathrm{cog}},w,{\vec{v}}_{\max}$, and ${\vec{v}}_{\min}$ are usually carefully selected according to the characteristics of the problem under investigation. Since these values cannot be determined analytically, their selection usually requires a massive amount of trials.

In this context, Fuzzy Self-Tuning PSO [23] adopts a radically different approach, which allows to avoid the settings problem by adding a Fuzzy Rule Based System (FRBS) to each particle. Specifically, Fuzzy Logic is used to dynamically (i.e., during the optimization) adjust the settings for each particle—independently from the other particles in the swarm—according to the performances of the particle and its distance with respect to the position of the current global best particle $\vec{g}$. Thanks to this approach, FST-PSO does not require any user settings and can outperform the classic PSO (and many competitor algorithms) in several benchmark [23] and real-world problems [25,26,27].

The pseudocode of the FST-PSO algorithm is shown in Algorithm 1: the algorithm begins by randomly placing the particles in the search space (line 1). Then, similarly to conventional PSO, the fitness function is evaluated for all particles, and the information about the current global and local bests are updated accordingly (lines 4–10). FST-PSO exploits the FRBS to perform multiple Sugeno inferences (line 14), according to each particle’s performance with respect to the previous iteration (line 12) and to its distance from the current global best in the swarm (line 13). Finally, the velocity and position of all particles are updated (lines 16–20), and the algorithm iterates until the budget of fitness evaluations is depleted. The best solution found by the swarm is returned as the result of the optimization (line 23).
**Algorithm 1:** Pseudocode of the FST-PSO algorithm.
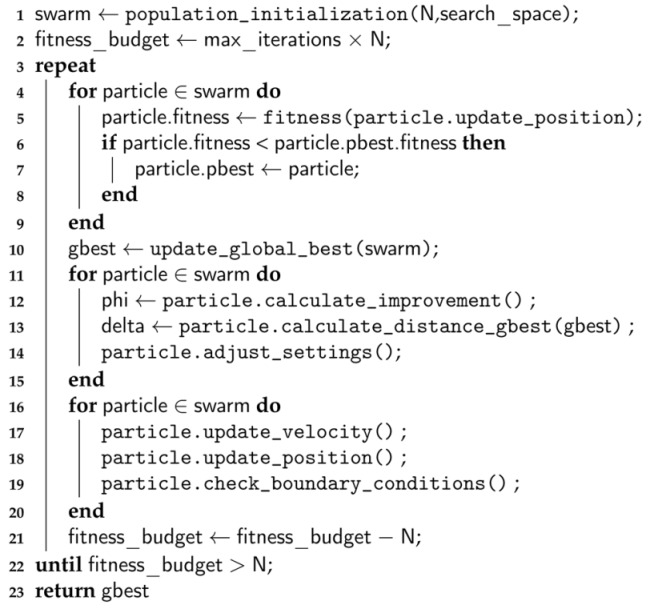


### 2.2. Fitness Landscape Surrogate Modeling with Fourier Filtering (surF)

The *discrete Fourier transform* (DFT) is one of the most useful mathematical tools to process signals and information represented in a discrete way [28]. Here we recall the definition of the DFT in the one-dimensional case, the way it is extended to higher dimensions, and how it can be employed in a process of “filtering” the fitness landscape to reduce the number of local minima.

Let f:[ℓ,u]→R be a fitness function for a given optimization problem, whose solutions belong to the interval [ℓ,u]⊆R. Suppose that we can sample *f* for ρ times at regular intervals, obtaining a set of fitness values $f\left(x_{0}\right),\ldots ,f\left(x_{\rho -1}\right)$, where $|x_{k}-x_{k+1}|=\Delta x$ for 0≤k<ρ−1. Stated otherwise, the ρ points $x_{0},\cdots ,x_{\rho -1}$ are all equispaced with distance Δx in the interval [ℓ,u]. The idea behind the DFT is that each sampled fitness value $f\left(x_{0}\right),\ldots ,f\left(x_{\rho -1}\right)$ can be represented as a weighted sum of frequencies, in the following way:

$$\begin{array}{ccc} f\left(x_{k}\right)=\sum_{m=0}^{\rho -1}a_{m}e^{-2\pi i\frac{mk}{\rho }} & \; & \mathrm{for}\;0\leq k<\rho . \end{array}$$

This means that the ρ coefficients $a_{0},\ldots ,a_{\rho -1}$ in the frequency domain are all that is needed to recover $f\left(x_{0}\right),\ldots ,f\left(x_{\rho -1}\right)$. Intuitively, each $a_{m}$ is the coefficient corresponding to a certain frequency, thus we can freely move from a collection of ρ points $f\left(x_{0}\right),\ldots ,f\left(x_{\rho -1}\right)$ in space (i.e., R, in this case), to a collection of ρ coefficients $a_{0},\ldots ,a_{\rho -1}$ for the frequencies and vice versa, via the inverse transformation called the *inverse* DFT.

Since applying the DFT first and then the inverse DFT corresponds to the identity function, we carry out an intermediate transformation step, which consists in filtering all frequencies above a certain threshold. This step, when operating in the frequency domain, simply consists in setting to zero all but the first γ coefficients, for some γ∈{0,⋯,ρ−1}, which correspond to the highest frequencies. Namely, we only consider the coefficients $a_{0}^{\prime },\ldots ,a_{\rho -1}^{\prime }$, where $a_{0}^{\prime }=a_{0},\ldots ,a_{\gamma -1}^{\prime }=a_{\gamma -1}$, and $a_{\gamma }^{\prime }=0,\ldots ,a_{\rho -1}^{\prime }=0$. When applying the inverse DFT to the set of coefficients $a_{0}^{\prime },\ldots ,a_{\rho -1}^{\prime }$, the resulting set of points $y_{0},\ldots ,y_{\rho -1}$ is a “smoothed” version of the starting set of points $f\left(x_{0}\right),\ldots ,f\left(x_{\rho -1}\right)$.

We can interpret the points $y_{0},\ldots ,y_{\rho -1}$ as those obtained via a function $\tilde{f}:\left[\ell ,u\right]\to R$ that is “smoother” than *f*, namely, $y_{0}=\tilde{f}\left(x_{0}\right),\ldots ,y_{\rho -1}=\tilde{f}\left(x_{\rho -1}\right)$. By initially employing $\tilde{f}$ as the fitness function in our optimization process we might be able to avoid local minima, while preserving the location of the global optima. This is, indeed, an assumption that might be or might not be satisfied by the specific fitness function under examination. We provide some examples and a discussion of this matter in Section 3.

Since we only have a sample of ρ values of $\tilde{f}$, to actually obtain a function defined on the entire interval [ℓ,u] it is necessary to interpolate between the points $\tilde{f}\left(x_{0}\right),\ldots ,\tilde{f}\left(x_{\rho -1}\right)$. This can be simply performed by a linear interpolation (i.e., connecting the points with segments), thus obtaining a piecewise linear function. Therefore, a sequence of steps to find the global optimum of an unknown function $f:\left[\ell ,u\right]\to R^{+}$ consists in: sampling *f* in a number ρ of equally spaced points; applying the DFT and reduce the number of coefficients; filtering out the higher frequencies; applying the inverse DFT to obtain a “smoothed” version of *f*—that is, its *surrogate model* defined by the function $\tilde{f}$—on which an optimization method can be applied. The resulting optima of $\tilde{f}$ found by the optimization method can then be “translated back” to *f*, with the assumption that, for some classes of functions, the global optima of $\tilde{f}$ will be the same or close to the global optima of *f*.

The same idea can be extended to any number of dimensions. Let the fitness function be defied as $f:\prod_{d=1}^{D}\left[\ell_{d},u_{d}\right]\to R^{+}$. For the sake of exposition, we assume that all intervals $\left[\ell_{d},u_{d}\right]$ are equal, so that we can rewrite the fitness function as $f:{\left[\ell ,u\right]}^{D}\to R^{+}$. Since we are working in a *D*-dimensional space, instead of sampling ρ equispaced points we will sample $\rho^{D}$ points, which are all equally spaced in a *D*-dimensional grid. The DFT can be extended into *D* dimensions, obtaining $\rho^{D}$ coefficients for the frequencies. For example, in the two-dimensional case there will be $\rho^{2}$ points $f\left({\vec{x}}_{1,1}\right),\ldots ,f\left({\vec{x}}_{1,\rho -1}\right),\ldots ,f\left({\vec{x}}_{\rho -1,1}\right),\ldots ,f\left({\vec{x}}_{\rho -1,\rho -1}\right)$, from which $\rho^{2}$ coefficients are obtained by the two-dimensional DFT: $a_{1,1},a_{1,2},\ldots ,a_{\rho -1,\rho -1}$. Then, we can keep only the $\gamma^{2}$ coefficients $a_{i,j}$ with 0≤i,j<γ corresponding to the lower frequencies. In *D* dimensions, the number of coefficients to keep is $\gamma^{D}$. As before, by performing the inverse DFT, we obtain a set of $\rho^{D}$ points from a function $\tilde{f}$, which can be interpreted as *f* where the higher frequency components were removed. The optimization can then proceed on the surrogate model $\tilde{f}$, at least initially.

One last point to notice is that, in order to perform the DFT, we need to sample the function *f* in $\rho^{D}$ points, which might not be always feasible, especially when computing *f* is resource-intensive. To mitigate this problem, we propose the following additional approximation:

1. a set $A=\{{\vec{x}}_{0},\cdots ,{\vec{x}}_{\sigma }\}$ of σ points, with $\sigma \ll \rho^{D}$, is defined by sampling *f* uniformly in ${\left[\ell ,u\right]}^{D}$;
2. a surrogate $\hat{f}$ of *f* is defined in the following way, for each $\vec{x}\in {\left[\ell ,u\right]}^{D}$:
  (a) if $\vec{x}$ is inside the convex hull of the points in *A*, then a triangulation of the points in *A* is constructed and the value of $\hat{f}\left(\vec{x}\right)$ is obtained by linear interpolation. For example, in two dimensions, $\vec{x}$ will be contained in a triangle defined by three points ${\vec{x}}_{i},{\vec{x}}_{j},{\vec{x}}_{k}\in A$, and $\hat{f}\left(\vec{x}\right)$ will be a linear combination of $f({\vec{x}}_{i})$, $f({\vec{x}}_{j})$, and $f({\vec{x}}_{k})$;
  (b) if $\vec{x}$ is outside the convex hull of the points in *A*, then $\hat{f}\left(\vec{x}\right)=f\left({\vec{x}}^{\prime }\right)$, where ${\vec{x}}^{\prime }\in A$ is the point in *A* that is nearest to $\vec{x}$.

So doing, the $\rho^{D}$ sampled points from function $\hat{f}$ can be obtained in a more efficient way, since *f* is only evaluated σ times. Actually, the function $\tilde{f}$ obtained by filtering the higher frequencies will actually be a smoothed version of $\hat{f}$ and not directly of *f*. The rationale of surF is exactly to get a pointer to *f* and return a pointer to $\tilde{f}$, which can be directly exploited in global optimization algorithms.

The pseudocode of surF is provided in Algorithm 2. The input of the algorithm is the original fitness function and its search space, along with the actual values for σ, ρ and γ. The fitness function is randomly sampled σ times in the search space (line 2), and this information is used to create the interpolation grid with step ρ (line 3). The interpolated grid is fed to the DFT to extract the Fourier coefficients (line 4). The first γ coefficients are conserved, while the rest is filtered out by setting the values to 0 (lines 5–8). Finally, the filtered coefficients are processed with inverse DFT to produce a pointer to the surrogate model, which is returned as output of the algorithm.
**Algorithm 2:** Pseudocode of the surF algorithm.
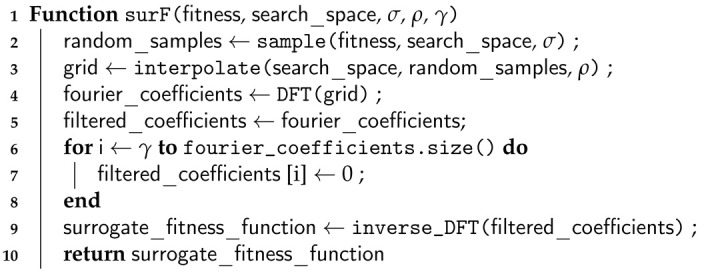


### 2.3. The Search on the Smoothed Landscape: Coupling surF with FST-PSO (F3ST-PSO)

Once the surrogate model of the fitness landscape is defined, it can be used as an actual fitness function to perform the optimization. The advantages provided by this approach are manifold:

- the surrogate model represents a smoothed version of the original fitness landscape, whose “smoothness” can be tuned by means of the γ hyperparameter;
- the evaluation of a candidate solution, using the surrogate model, requires a small computational effort. Notably, the latter can be far smaller than the evaluation of the original fitness function, especially in the case of real-world engineering or scientific problems (e.g., parameter estimation of biochemical systems [29], integrated circuits optimization [14], vehicle design [15]);
- even if an optimization performed on the surrogate model (e.g., using FST-PSO) does not require any evaluation of the original fitness function, it can provide useful information about the fitness landscape and the likely position of optimal solutions;
- the information about the optimal solutions found on the surrogate model can be used for a new optimization, leveraging the original fitness function.

We propose a novel methodology, named Fourier Filtering Fuzzy Self-Tuning Particle Swarm Optimization (F3ST-PSO), which couples surF and FST-PSO and works by performing the following operations:

1. a part of the fitness evaluations budget is reserved for surF to randomly sample the search space and create the surrogate model;
2. a preliminary optimization on the surrogate model is performed with FST-PSO, to identify an optimal solution ${\vec{g}}^{≃}$;
3. a new FST-PSO instance is created, and ${\vec{g}}^{≃}$ is added to the initial random population;
4. a new optimization is performed, exploiting the original fitness function and using the remaining budget of fitness evaluations;
5. a new optimal solution ${\vec{g}}^{\mathrm{real}}$ is determined and returned as a result of the whole optimization.

**Figure 2:**
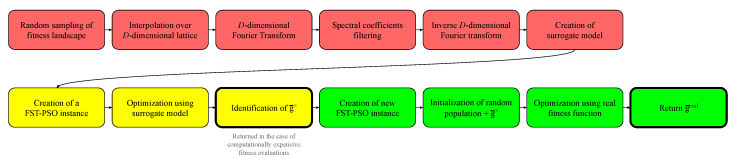
Detailed scheme of F3ST-PSO functioning. In the first phase (red boxes), the algorithm creates the surrogate model of the fitness landscape, by exploiting random sampling and Fourier filtering. The second phase (yellow boxes) consists in an optimization by means of FST-PSO over the surrogate model. In the third phase (green boxes), the best solution found is fed to a new FST-PSO optimization step over the real fitness function.

The complete functioning of F3ST-PSO is schematized in Figure 2. It is worth noting that, in the case of computationally expensive fitness functions, the algorithm can skip the points 3, 4, and 5 (i.e., the green boxes in Figure 2) and directly return ${\vec{g}}^{≃}$ as solution to the optimization problem.

### 2.4. Frequency of the Optimum Conjecture

Given a fitness function f:[ℓ,u]→R (the following discussion is for a single dimension but generalizes to any number of dimensions), let us assume that *f* has a unique global optimum o∈[ℓ,u]; it is then possible to define the *frequency of the optimum o*. Informally, the frequency of *o* is given by the minimum number of terms of the Fourier transform of *f* to keep in order to obtain an approximation $\hat{f}$ of *f* where *o* is still the global optimum. Stated otherwise, only a subset of the coefficients of the Fourier transform of *f* are necessary to recover the global optimum.

Formally, let

$$\begin{array}{ccc} f\left(x\right)=\sum_{n=-\infty }^{+\infty }c_{n}e^{2\pi i\frac{nx}{u-\ell }} & \; & \mathrm{for}\;x\in [\ell ,u] \end{array}$$

be the function *f* written as a Fourier series, and let o∈[ℓ,u] be such that $o={\mathrm{argmin}}_{x\in [\ell ,u]}f\left(x\right)$ (i.e., here *o* represents the global minimum, the reasoning for the global maximum is symmetric). We say that *o* has frequency γ if the function $f_{\gamma }$ defined as:

$$\begin{array}{ccc} f_{\gamma }\left(x\right)=\sum_{n=-\gamma }^{+\gamma }c_{n}e^{2\pi i\frac{nx}{u-\ell }} & \; & \mathrm{for}\;x\in [\ell ,u], \end{array}$$

with $o_{\gamma }={\mathrm{argmin}}_{x\in [\ell ,u]}f_{\gamma }\left(x\right)$ is such that $o_{\gamma }=o$ and, for all $\gamma^{\prime }<\gamma$, $o_{\gamma^{\prime }}\neq o$. We can allow for *o* and $o_{\gamma }$ to not be equal but only at a distance at most equal to ε, for some ε>0: we say that the frequency of the optimum *o* with error ε is γ if $|o-o_{\gamma }|<\varepsilon$ and, for all $\gamma^{\prime }<\gamma$, $|o-o_{\gamma^{\prime }}|\geq \varepsilon$.

Notice that in this definition we do not use the idea of performing a sampling of *f*, or a sampling of an approximation of *f*, but we work directly with the function *f* and its Fourier transform. However, in practical cases *f* is not directly available, thus requiring some approximations and “tricks”.

It is interesting to notice that working on the approximation $f_{\gamma }$ of *f*, where the frequency of the optimum is γ, can be beneficial: while there is no restriction on the number of local optima in *f*, the fact that $f_{\gamma }$ is defined as a finite sum of frequencies means that the number of local optima in *f* is bounded above by an increasing function of γ, thus limiting the number of places where an optimization process might “get stuck” in $f_{\gamma }$.

One question that remains unanswered by the previous definition is *how* to find the frequency of the optimum. If the frequency is low enough, then using a low value of γ can be beneficial, since the number of local optima can be greatly reduced. On the contrary, if the frequency is high enough, then $f_{\gamma }$ for a low value of γ might not help in the optimization process, since the location of the global optimum is different in *f* and in $f_{\gamma }$. If the value of γ is high enough there might not be a reduction in the number of local optima, thus making the use of $f_{\gamma }$ not beneficial from an optimization point of view.

In fact, since we are truncating the Fourier series of *f* to a limited number of terms, if *f* has a global optimum at a high frequency but has only a limited number of local optima, it might be possible to *increase* the number of local optima in $f_{\gamma }$, making the problem more difficult to solve. Therefore, approximating *f* with $f_{\gamma }$ is expected to be more beneficial when the frequency of the global optimum is low enough that $f_{\gamma }$ actually decreases the number of local optima by smoothing *f*.

## 3. Results and Discussion

The aim of the tests presented in this section is twofold: first, we assess the actual capability of surF in creating surrogate models that are easier to explore, while preserving the specific features of the fitness landscape; second, we investigate the entire process to evaluate whether our methodology could be beneficial to solve the optimization problem. To this aim, we tested F3ST-PSO on the benchmark functions listed in Table 1, and on a subset of the CEC 2005 test suite. The Ackley, Alpine, Griewank, Michalewicz, Rastrigin, Schwefel, Shubert, and Vincent functions were selected because they represent excellent candidates to study the effectiveness of our approach, thanks to their structural characteristics (multimodal, rugged, and noisy).

In all tests that follow, we considered D=5 to limit the memory occupancy. Indeed, as the function $\hat{f}$ has to be sampled $\rho^{D}$ times, the requirements in terms of time and memory needed to store the samples and to compute the DFT increase exponentially with the number of dimensions, even if we sample the function *f* only σ times to obtain $\hat{f}$. The number of initial samples of the fitness landscape (i.e., the fitness evaluations) was set to σ=500, while the resolution of the grid used for the interpolation step was set to ρ=40 points. All tests were repeated 30 times to collect statistically significant results. In order to make quantitative comparisons between the different methods, we calculated the Average Best Fitness (ABF), i.e., the mean of the fitness values of the best solution found at each iteration, evaluated over the 30 runs.

surF and F3ST-PSO were implemented using Python 3.7.4, NumPy 1.17.3 [30], and SciPy 1.3.1 [31]. The Python implementation of the IEEE CEC 2005 benchmark suite was based on the optproblems library. Due to the exceptional computational effort required to perform all tests, the calculations were offloaded on SURFsara’s Cartesius supercomputer, equipped with 66 nodes based on 2×8-core 2.5 GHz Intel Xeon E5-2450 v2.

### 3.1. Generation of Surrogate Models by surF

Except for the Rosenbrock function, the other benchmark functions defined in Table 1 contain at least one trigonometric term, which was exploited on purpose to mimic noise or to introduce a large number of local minima in the landscape. One of the scopes of this work is to show that these fluctuations in the fitness values can be removed by means of Fourier filtering, yielding a smoother fitness landscape that, ultimately, leads to a simpler version of the original optimization problem.

**Figure 3:**
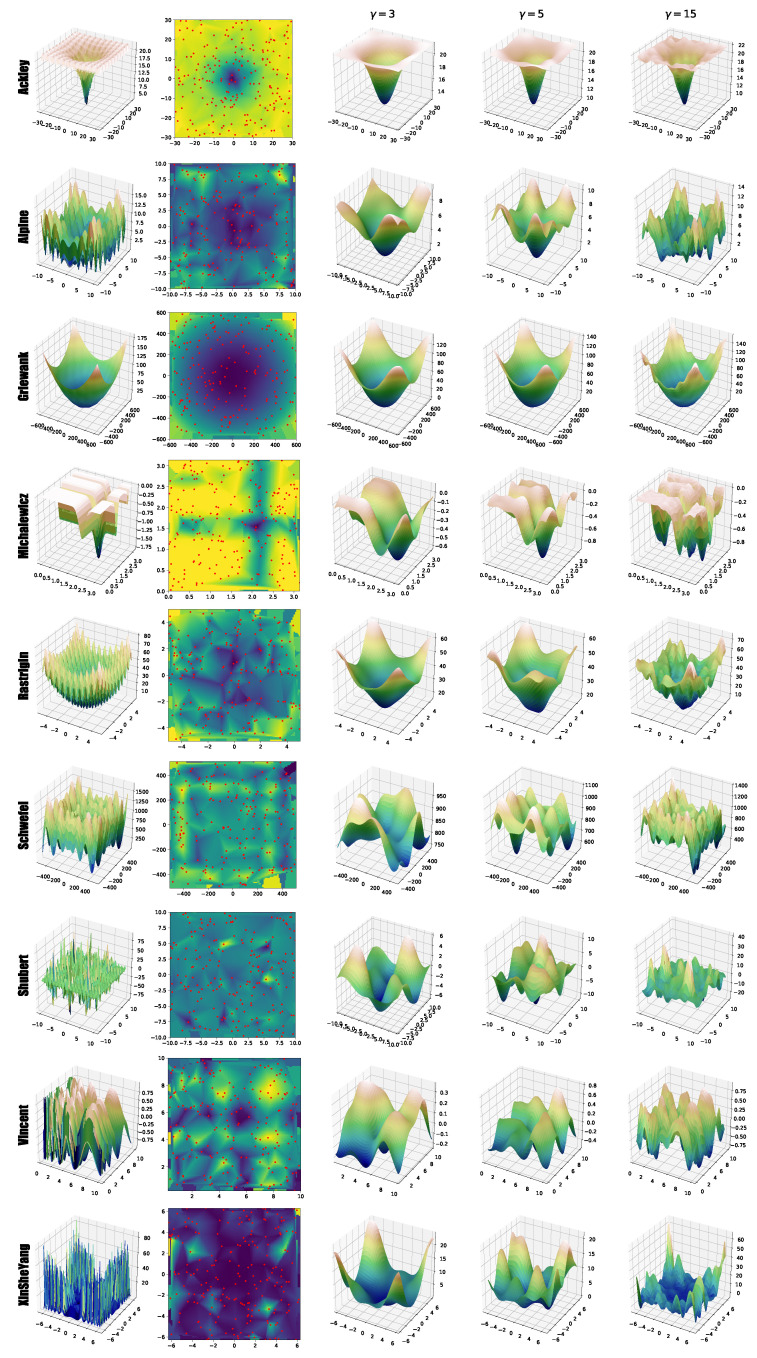
Examples of 2D surrogate models of the benchmark functions defined in Table 1, created by surF. The first column shows the original fitness landscape. The second column represents a random sampling of the fitness landscape, which is used to create the interpolation grid for the Fourier transform. The interpolation is shown as background color: dark/blue colors correspond to good fitness values, while bright/yellow colors correspond to bad fitness values. The third, fourth, and fifth columns represent the surrogate models, obtained by applying the inverse Fourier transform using γ=3, γ=5 and γ=15 coefficients, respectively.

In Figure 3, first column, we present the original fitness landscape for each benchmark function, while the last three columns show some examples of their 2D surrogate models, created using surF and adopting an increasing number of coefficients in the frequency domain (i.e., γ=3, γ=5 and γ=15). All plots were generated using σ=100 and ρ=200. For each benchmark function, we sampled the ρ=200 random points from the fitness landscape using a uniform distribution (as shown in the second column), exploiting a pseudorandom generator (Mersenne Twister [32]). These points were used in surF to create an interpolated grid, calculate a two-dimensional Fourier transform, filter the high-frequency components, and produce the surrogate model.

The results presented in Figure 3 show that, when using γ=3 (third column), the surrogate models of some benchmark functions are very similar to the original fitness landscape, with the exception of the high frequency perturbations (compare, e.g., the original fitness landscape of the Ackley function and its surrogate model). In particular, in many cases the position of the global minimum is conserved even when using such a small number of coefficients. We then repeated the process using γ=5 and γ=15 coefficients (fourth and fifth column, respectively), to show that a larger number of coefficients can correctly reproduce the high frequency components of the original fitness landscape (see, e.g., the Michalewicz and Rastrigin functions).

### 3.2. Optimization of Benchmark Functions by F3ST-PSO

**Figure 4:**
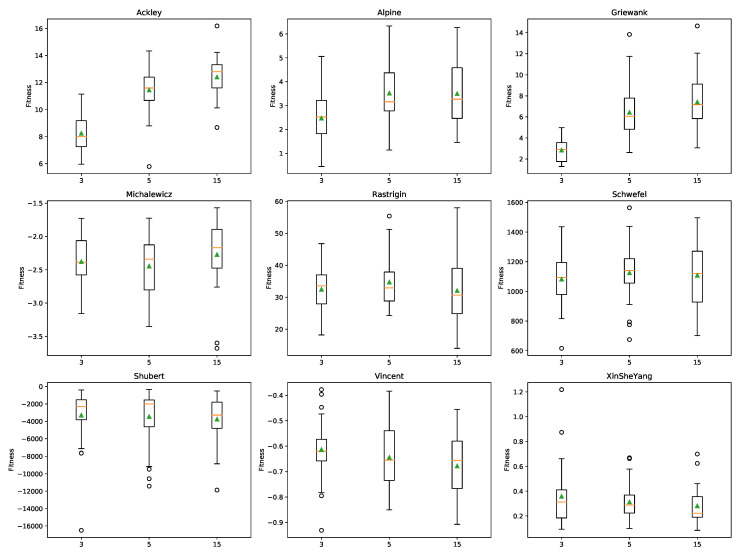
Boxplots of the fitness values distribution of the best individuals ${\vec{g}}^{≃}$ found by FST-PSO at the last iteration on the surrogate models, exploiting γ=3, γ=5 and γ=15 coefficients (*x*-axis). The orange line and the green triangle denote the median and the mean, respectively.

surF was applied to create the surrogate models of the fitness landscapes of the nine benchmark functions, considering γ=3, γ=5 and γ=15 coefficients. We then performed an optimization phase with FST-PSO on the surrogate models, using 150 particles and 1000 iterations. Note that these optimizations did not require any evaluation of the original fitness function, except for the initial random sampling of the fitness landscape that was carried out by surF. Figure 4 shows the boxplots of the fitness values distributions of the best solutions ${\vec{g}}^{≃}$ found by FST-PSO for the optimization of different benchmark functions, whose surrogate models were created with different γ values. Note that the fitness values were calculated using the actual fitness function.

These results show that in the case of the Ackley, Alpine, Griewank, and Michalewicz functions, the best performances were obtained with the surrogate models using a few coefficients. Stated otherwise, increasing the detail of the surrogate model does not necessarily lead to better results and might even affect the optimization performances. This phenomenon could be due to the higher level of multimodality and ruggedness introduced into the surrogate model by using higher frequency components. This is particularly evident in the case of the Alpine function, when comparing the plots of the surrogate models generated with γ=3 and γ=15 (Figure 3). By using 15 coefficients, many local minima of the original fitness landscape are maintained in the surrogate model. We also observed that creating a surrogate model of a function characterized by a logarithmic component, as in the case of the Vincent function, can bend the resulting fitness landscape so that, despite being smoother than its original counterpart, it completely loses its specific features. Finally, a different effect can be noticed in the case of the Michalewicz benchmark function, where the plateaus of the original fitness landscape are introduced into the surrogate model as uneven areas, making the optimization problem harder to solve. We believe that this might be related to the Gibbs phenomenon of the Fourier series, whereby cutting the high frequencies of the fitness function introduces ringing artifacts [33].

**Figure 5:**
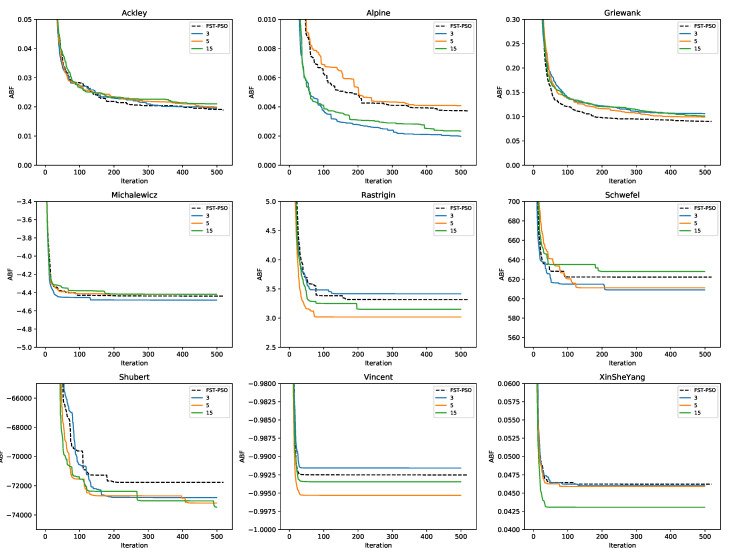
Convergence plot showing the performance of FST-PSO (black dashed line) against F3ST-PSO (blue, orange, and green solid lines correspond to the use of γ=3, 5, and 15 coefficients in surF, respectively). The plots show that FST-PSO can perform 20 additional iterations compared to F3ST-PSO, since the construction of the surrogate model “consumes” 500 fitness evaluations during the initial random sampling.

We then compared the performances of F3ST-PSO—when all five phases of the methodology are executed—against standard FST-PSO (i.e., with no previous application of surF). The settings used for these tests are reported in Table 2. The results of this comparison are shown in Figure 5, where FST-PSO is denoted by the black dashed line, while F3ST-PSO is denoted by solid blue, orange, and green lines for γ=3, γ=5 and γ=15, respectively. According to these results, F3ST-PSO always showed better performances in the case of the Shubert function.

In the case of the Rastrigin and Vincent functions, F3ST-PSO outperformed FST-PSO by using γ=5 and γ=15. In the case of the Michalewicz function, higher values of γ seem to lead to worse results, arguably due to the increased number of local minima. FST-PSO performed similar to F3ST-PSO in the case of the Ackley function and yielded better results in the case of the Griewank function.

Altogether, these results suggest that the preliminary optimization phase on the surrogate model can actually have a positive impact on the identification of the global optimum.

Finally, in relation to the frequency of the optimum conjecture described in Section 2.4, we observed that F3ST-PSO was outperformed by FST-PSO in the case of the Rastrigin and Vincent functions, for γ=3. This situation can be explained by the fact that the frequency of the optimum might be higher than 3, implying that the initial optimization phase did not provide any optimal solution for the second optimization phase.

### 3.3. Optimization of the CEC 2005 TEST suite by F3ST-PSO

**Figure 6:**
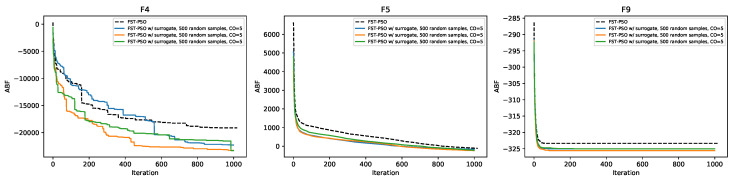
Convergence plot showing the performance of FST-PSO (black dashed line) against F3ST-PSO (blue, orange, and green solid lines correspond to the use of γ=3, 5, and 15 coefficients in surF, respectively). The plots correspond, from left to right, to the results on the benchmark functions F4, F5, and F9 of the CEC 2005 suite.

We investigated the performances of F3ST-PSO on more complex fitness functions, by using the CEC 2005 test suite. The settings used for these tests are shown in Table 3. Specifically, we considered the subset of functions that are defined for problems with D=5 (i.e., F1, F2, F4, F5, F6, F9, F13, F15). Functions F1, F2, F6, F13, and F15 were very simple to solve, and both F3ST-PSO and FST-PSO immediately converged to the optimal solutions with similar performances (data not shown). On the contrary, the two algorithms showed significant differences in the optimization of functions F4, F5, and F9, as shown in Figure 6.

**Figure 7:**
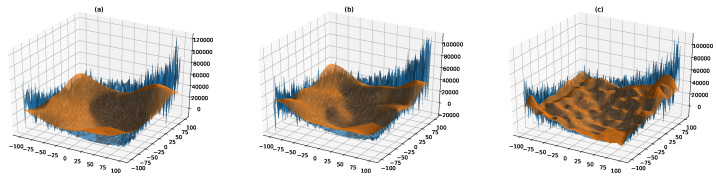
Surrogate models of function F4 using γ=3, γ=5 and γ=15 coefficients (plots (**a**), (**b**), and (**c**), respectively). By removing the higher components of the Fourier transform, the random noise is reduced, so that the fitness landscape becomes smoother and easier to explore (orange surface), while retaining the general characteristics of the original problem (blue surface).

In particular, the case of function F4 is worth discussing. This benchmark function represents a shifted version of the Schwefel’s optimization problem [34], with additional noise [35]. The (multiplicative) noise applied to the function is calculated by taking the absolute value of scaled normally distributed random numbers:

$$F4\left(\vec{x}\right)=\left(\sum_{d=1}^{D}\left(\sum_{j=1}^{d}z_{j}\right)\right)\cdot (1+0.4\cdot |N\left(0,1\right)\left|\right),\;\vec{z}=\vec{x}-\vec{o},$$

where $\vec{o}$ is the shifted position of the global optimum. Due to noise, two evaluations of function F4 using the same candidate solution return different fitness values, misleading all the approaches that are purely based on gradient descent and, potentially, also swarm intelligence techniques like PSO. On the contrary, surF can produce a smoother and easier version of the fitness landscape (Figure 7). Thanks to this smoothing, F3ST-PSO actually led to better performances, with a final ABF lower than FST-PSO regardless of the γ value used (Figure 6, left panel).

## 4. Conclusions

In this work we presented surF, a novel methodology for the creation of surrogate models of fitness landscapes by means of Fourier filtering. Differently from similar surrogate modeling approaches—which are generally adopted to mitigate the computational effort of fitness evaluations—surF also allows the user to select the level of “ruggedness” of the search space by setting a specific hyperparameter γ, which gives control over the number of low-frequency harmonics used for the inverse Fourier transform. We tested surF using nine well known benchmark functions characterized by multimodality, showing that it can be effective in several cases to smooth out the fitness landscape, preserving the global optimum of the function. However, some fitness landscapes can be characterized by pathological circumstances, in which the optimum cannot be reproduced using low frequency components: in these cases, better results can be obtained by using higher values for γ.

We then coupled surF to the FST-PSO settings-free global optimization metaheuristics, thus creating a novel algorithm named F3ST-PSO. This dual-phased methodology begins by performing a first optimization phase on the surrogate model; then, the global optimum identified in the surrogate (notably, without performing any fitness evaluation) is added to a new random population that undergoes a second optimization phase on the real model. We showed that F3ST-PSO can outperform a pure optimization run made by FST-PSO; still, it is worth noting that the second optimization phase can be skipped in the presence of computationally expensive fitness evaluations.

The main inconvenience of surF, at the moment, is the interpolation of the random samples of the fitness function over a *D*-dimensional grid, which is necessary to evaluate the Fourier transforms and is characterized by a very high time and space complexity. Specifically, if ρ partitions are considered for each axis of the search space, then a grid of $\rho^{D}$ interpolated points must be calculated, making the naïve implementation infeasible for high-dimensional optimization problems. As a future development, we will investigate alternative approaches for the effective calculation of Fourier transforms on high-dimensional search spaces with a less-than-exponential complexity, possibly removing the need for the ρ hyperparameter.

A second drawback of surF is the frequency of the optimum, i.e., the possibility that some optimal solutions cannot be represented using low values for γ. This hyperparameter plays a relevant role in the optimization and, of course, the optimal selection of its value is strictly problem dependent. It might also be the case that a craftful manipulation of the fitness landscape (notably, by means of Dilation Functions [36]) might reveal such optima even using low frequency components. We will investigate this possibility, in particular, in the domain of stochastic biochemical systems that are characterized by very noisy fitness landscapes [37] and a log-uniform distribution of the components of optimal solutions within the search space [25].

Finally, the sampling of the search space exploited by surF is based on pseudorandom sequences. Alternative sampling methods could be exploited (e.g., quasirandom sequences [38], cellular automata [39], latin hypercubes [40]), and possibly lead to better (or perhaps biased) approximations. We will investigate the impact of these initial sampling methods on Fourier-based surrogate modeling, to the aim of further improving the optimization task.

The source code of surF and some usage examples are available on GITHUB at the following address: https://github.com/aresio/surF. The surF Python package can be installed from the PyPI repository by typing: pip install surfer.

## Figures and Tables

**Table 1:** Benchmark functions.

Function	Equation	Search Space	Value in Global Minimum
Ackley	$f_{\mathrm{Ack}}\left(\vec{x}\right)=20+e-20\exp\left(-0.2\sqrt{\frac{1}{D}\sum_{d=1}^{D}x_{d}^{2}}\right)-$ $\exp(\frac{1}{D}\sum_{d=1}^{D}\cos\left(2\pi x_{d}\right))$	${\left[-30,30\right]}^{D}$	$f_{\mathrm{Ack}}\left(\vec{0}\right)=0$
Alpine	$f_{\mathrm{Alp}}\left(\vec{x}\right)=\sum_{d=1}^{D}\left|x_{d}\sin\left(x_{d}\right)+.1x_{d}\right|$	${\left[-10,10\right]}^{D}$	$f_{\mathrm{Alp}}\left(\vec{0}\right)=0$
Griewank	$f_{\mathrm{Gri}}\left(\vec{x}\right)=\frac{1}{4000}\sum_{d=1}^{D}x_{d}^{2}-\prod_{d=1}^{D}\cos\left(\frac{x_{d}}{\sqrt{d}}\right)+1$	${\left[-600,600\right]}^{D}$	$f_{\mathrm{Gri}}\left(\vec{0}\right)=0$
Michalewicz	$f_{\mathrm{Mic}}\left(\vec{x}\right)=-\sum_{d=1}^{D}\sin\left(x_{d}\right)\sin^{2k}\left(\frac{dx_{d}^{2}}{\pi }\right)$, k=10 in this work	${\left[0,\pi \right]}^{D}$	$f_{\mathrm{Mic}}\left(0,0\right)=-1.801$ $f_{\mathrm{Mic}}\left(0,0,0,0,0\right)=-4.687$
Rastrigin	$f_{\mathrm{Ras}}\left(\vec{x}\right)=10D+\sum_{d=1}^{D}\left(x_{d}^{2}-10\cos\left(2\pi x_{d}\right)\right)$	${\left[-5.12,5.12\right]}^{D}$	$f_{\mathrm{Ras}}\left(\vec{0}\right)=0$
Rosenbrock	$f_{\mathrm{Ros}}\left(\vec{x}\right)=\sum_{d=1}^{D-1}\left[100{\left(x_{d}^{2}-x_{d+1}\right)}^{2}+{\left(x_{d}-1\right)}^{2}\right]$	${\left[-5,10\right]}^{D}$	$f_{\mathrm{Ros}}\left(\vec{1}\right)=0$
Schwefel	$f_{\mathrm{Sch}}\left(\vec{x}\right)=418.9829D-\sum_{d=1}^{D}x_{d}\sin(\sqrt{|x_{d}\left|\right)}$	${\left[-500,500\right]}^{D}$	$f_{Sch}\left(\vec{420.9687}\right)=0$
Shubert	$f_{Shu}\left(\vec{x}\right)=\prod_{d=1}^{D}\left(\sum_{i=1}^{5}i\cos\left[\left(i+1\right)x_{d}+i\right]\right)$	${\left[-10,10\right]}^{D}$	Many global minima, whose values depend on *D*
Vincent	$f_{\mathrm{Vin}}\left(\vec{x}\right)=\sum_{d=1}^{D}\sin\left(10\log\left(x_{d}\right)\right)$	${\left[0.25,10\right]}^{D}$	$f_{\mathrm{Vin}}\left(\vec{7.706281}\right)=-D$
Xin-She Yang n.2	$f_{\mathrm{Xin}}\left(\vec{x}\right)=\sum_{d=1}^{D}\left|x_{d}\right|{\left[\exp\left(\sum_{d=1}^{D}\sin\left(x_{d}^{2}\right)\right)\right]}^{-1}$	${\left[-2\pi ,2\pi \right]}^{D}$	$f_{\mathrm{Xin}}\left(\vec{0}\right)=0$

**Table 2:** Settings used for the comparison of performances between F3ST-PSO and FST-PSO, considering the benchmark functions with D=5.

Setting	Value
Fitness evaluations budget	13,000
σ	500
ρ	40
γ values tested	3, 5 and 15
Swarm size F3ST-PSO	25
Iterations F3ST-PSO	500
Swarm size of FST-PSO	25
Iterations FST-PSO	520

**Table 3:** Settings used for the comparison of performances between F3ST-PSO and FST-PSO, considering the functions F4, F5, and F9 of the CEC 2005 suite, with D=5.

Setting	Value
Fitness evaluations budget	25,500
σ	500
ρ	40
γ values tested	3, 5 and 15
Swarm size F3ST-PSO	25
Iterations F3ST-PSO	1000
Swarm size of FST-PSO	25
Iterations FST-PSO	1020

## Abbreviations

The following abbreviations are used in this manuscript:

DFT	Discrete Fourier Transform
EC	Evolutionary Computation
FRBS	Fuzzy Rule Based System
FST-PSO	Fuzzy Self-Tuning Particle Swarm Optimization
F3ST-PSO	Fourier Filtering Fuzzy Self-Tuning Particle Swarm Optimization
PSO	Particle Swarm Optimization
surF	Surrogate modeling with Fourier filtering
